# Effects of Statin Therapy on Glycemic Control and Associated Factors Among Type 2 Diabetes Mellitus Patients in Northeastern Tanzania: A Retrospective Cohort Study

**DOI:** 10.1155/jdr/6626154

**Published:** 2025-08-01

**Authors:** Daniel P. Mujuni, Kajiru G. Kilonzo, Abid M. Sadiq, Norman J. Kyala, Philip C. Makupa, Sweetness N. Laizer, Elifuraha W. Mkwizu, Furaha S. Lyamuya, Elichilia R. Shao, Erick A. Mboya, Nyasatu G. Chamba

**Affiliations:** ^1^Department of Internal Medicine, KCMC University, Moshi, Tanzania; ^2^Department of Internal Medicine, Kilimanjaro Christian Medical Centre, Moshi, Tanzania; ^3^Department of Preventive Services, Ministry of Health, Dodoma, Tanzania; ^4^Department of Clinical Research, Kilimanjaro Clinical Research Institute, Moshi, Tanzania; ^5^Department of Epidemiology and Statistics, Muhimbili University of Health and Allied Sciences, Dar es Salaam, Tanzania; ^6^Department of Epidemiology, KCMC University, Moshi, Tanzania

**Keywords:** dyslipidemia, glycated hemoglobin (HbA_1c_), glycemic control, statins, Tanzania, Type 2 diabetes mellitus

## Abstract

**Introduction:** Statins have been implicated in poor glycemic control among patients with diabetes mellitus (DM), prompting the US Food and Drug Administration (FDA) to update warning labels on all statins to reflect the risk of increased blood glucose levels. However, few studies from sub-Saharan Africa have assessed this concern. This study investigated the effects of statins on glycemic control among patients with Type 2 diabetes mellitus (T2DM) in Kilimanjaro, northeastern Tanzania.

**Materials and Methods:** This was a hospital-based retrospective cohort study evaluating changes in glycated hemoglobin (HbA_1c_) at 1–3, 7–12, and 19–24 months, as the primary outcome, comparing statin users and nonusers among T2DM patients attending DM clinic at Kilimanjaro Christian Medical Centre in Tanzania. Binomial regression models were fitted to calculate adjusted risk ratios for independent predictors of a ≥ 0.2% rise in HbA_1c_, with statistical significance set at *p* < 0.05.

**Results:** Out of 122 patients, 51 (41.8%) were on statin therapy. Among these, 46 (90.2%) were prescribed atorvastatin. Statin users had an increase of mean HbA_1c_ from 10.6% ± 2.7% at baseline compared to 11.6% ± 2.8% at 1–3 months (*p* = 0.114), followed by a decrease to 10.1% ± 2.2% at 7–12 months (*p* = 1.0), and 10.0% ± 2.5% at 19–24 months (*p* = 1.0). However, atorvastatin users (*n* = 46) had a significant increase of mean HbA_1c_ from 10.7% ± 2.8% at baseline compared to 11.9% ± 2.7% at 1–3 months (*p* = 0.04). In contrast, nonstatin users had a consistent and significant decrease in HbA_1c_ from 11.3% ± 2.8% at baseline compared to 9.7% ± 2.2% at 1–3 months (*p* = 0.001), to 9.7% ± 2.6% at 7–12 months (*p* = 0.011), and to 9.3% ± 2.2% at 19–24 months (*p* = 0.001).

**Conclusion:** Statin therapy among patients with T2DM was associated with short-lived worsening of glycemic control at 1–3 months posttherapy.

## 1. Introduction

Statins, or 3-hydroxy-3-methylglutaryl coenzyme A (HMG-CoA) reductase inhibitors, have been widely used since 1982 to manage dyslipidemia and reduce the risk of atherosclerotic cardiovascular diseases (ASCVDs) [1]. Statins are among the most prescribed lipid-lowering agents due to their proven efficacy, safety, and tolerability [2]. However, statins have been associated with adverse effects such as myopathy, and growing evidence suggests a potential impact on glycemic control [3–5].

Statins are broadly classified into two types based on their solubility: lipophilic (e.g., atorvastatin) and hydrophilic (e.g., rosuvastatin) [6]. Both types inhibit HMG-CoA reductase, the rate-limiting enzyme in hepatic cholesterol synthesis [7]. This inhibition upregulates low-density lipoprotein cholesterol (LDL-C) receptors on hepatocytes, thereby enhancing LDL-C clearance from plasma and promoting cholesterol conversion to bile acids [8]. Beyond lipid-lowering, statins have additional benefits in preventing ASCVD by maintaining plaque stability, reducing inflammation, preventing thrombus formation, and inhibiting endothelial dysfunction, particularly in patients with T2DM [1, 9].

Despite strong evidence supporting the use of statins on preventing ASCVD [10, 11], their impact on glycemic control remains controversial [12]. Some studies report significant improvement on glucose levels [13], while others raise concerns about increased risk of worsening glycemic control, and new onset T2DM [14–16]. Notably, the JUPITER trial suggested that statins may modestly increase blood glucose levels and HbA_1c_ potentially by impairing insulin secretion. However, the overall benefits on ASCVD outweighed the risks of DM [3, 17, 18]. In 2012, the US FDA issued a label update for statins, warning of possible increase in fasting blood glucose (FBG) and HbA_1c_ levels [19].

While statins offer well-established cardiovascular benefits [20], their impact on glycemic control is a topic of debate [14]. Hyperglycemia contributes to endothelial damage through nonenzymatic glycation of proteins, a key initiating step in the development of atherosclerosis, and an independent risk factor for both micro- and macrovascular complications [21]. Notably, the link between poor glycemic control and DM-nephropathy remains unclear, and it is unclear whether statins–despite their pleotropic benefits—modify this risk [22, 23].

A 1% increase in HbA_1c_ is associated with a 14% higher risk of myocardial infarction and a 21% increase in diabetes-related deaths [24]. The burden of T2DM complications and premature deaths is disproportionately high in low- and lower-middle-income countries, where three out of four adults have T2DM. In 2021 alone, these regions accounted for ~5.3 million diabetes-related deaths [25]. In Tanzania, limited resources for primary and secondary prevention contributes to a rising disease prevalence—from 472,900 cases in 2011 to 2.9 million cases in 2021—with an estimated prevalence of 12.3% [25], and approximately 50% of people with T2DM had at least one diabetes-related complication [26].

Most literature on statins and glycemic control originates from high-income countries, with limited data from sub-Saharan Africa. Genetic polymorphisms between the two populations—particularly apolipoprotein E variants *ε*_4_ and *ε*_3_, which are more common in people of African ancestry—may influence cholesterol metabolism, adiponectin levels, statin responsiveness, and insulin sensitivity [27, 28], highlighting the need for population-specific research. In Tanzania, the effect of statins on glycemic control remains unclear.

This study aimed to assess the effects of statins on glycemic control in T2DM patients at KCMC hospital in Tanzania, in consideration of the type, dose, and duration of statin therapy, as well as other associated risk factors in a routine primary care setting.

## 2. Materials and Methods

### 2.1. Study Design and Setting

This was a hospital-based retrospective cohort study undertaken between October 2023 and May 2024, among patients with T2DM who attended the diabetes clinic at KCMC hospital in northeastern Tanzania, between January 2019 and May 2024.

Patients were enrolled based on predefined eligibility criteria and followed up for 24 months. Inclusion criteria included adults aged ≥ 18 years with a confirmed diagnosis of T2DM for at least 12 months before enrollment, who had not received statin therapy in the preceding 6 months and maintained the same type of statin throughout the study period. T2DM diagnosis was confirmed using at least two readings of either FBG ≥ 7 mmol/L, random blood glucose (RBG) ≥ 11.1 mmol/L, or HbA_1c_ ≥ 6.5% [10].

Exclusion criteria excluded patients with estimated glomerular filtration rate (eGFR) below 45 mL/min/1.73 m^2^, due to potential glycemic improvement [29]; systemic steroid use and polycystic ovarian syndrome, both impair insulin sensitivity and elevate plasma glucose; pregnancy, hemoglobin < 11 g/dL, or human immunodeficiency virus (HIV), all are high turnover states affecting HbA_1c_ accuracy [10]; and chronic liver disease due to impaired statin elimination and risk of drug accumulation [30].

Based on existing literature the mean change in HbA_1c_ was 0.35% in the exposed, this study expected a mean change in HbA_1c_ of 0.2% in the exposed, with standard deviation (SD) of 2 [3, 31]. A significance level (*Z* constant) of 0.05 was used. Accounting for 25% attrition, the study determined the total sample size of 128 would suffice with the power of the study above 80% [32].

This study was initially planned for two years 2021–2023, but due to missing data of key variables, such as HbA_1c_ and lipid profile during follow-up, enrolment was extended from 2019–2024. Data from the Preventive Treatment of Latent Tuberculosis Infection in People with Diabetes Mellitus (PROTID) [33] were used as a second data source for quality control and missing HbA_1c_ at 1–3 and 7–12 months for 38 of the patients who concurrently had been attending the DM clinic at KCMC.

### 2.2. Operational Definitions

Hypertension was defined as systolic blood pressure (SBP) ≥ 140 mmHg and/or diastolic blood pressure (DBP) ≥ 90 mmHg measured on two separate occasions of at least 6 h apart [34].

Dyslipidemia was defined as one or more of the following lipid abnormalities: total cholesterol > 5.2 mmol/L, high − density lipoprotein cholesterol (HDL‐C) ≤ 1.0 mmol/L, and LDL‐C > 3.4 mmol/L [11].

CKD was defined as eGFR < 45 mL/min/1.73 m^2^ calculated using the modification of diet in renal disease (MDR) equation [35].

Body mass index (BMI) was categorized as underweight (< 18.5 kg/m^2^), normal (18.5–24.9 kg/m^2^), overweight (25.0–29.9 kg/m^2^), or obese (≥ 30 kg/m^2^) [36].

Anemia, for the purpose of this study, was defined as hemoglobin < 11 g/dL [37].

Chronic liver disease was defined as a progressive deterioration of liver synthetic and metabolic functions for more than 6 months [38].

### 2.3. Participant Recruitment

Participants were retrospectively identified using the electronic hospital data management system and the diabetes clinic attendance registry. A total of 8051 patients with T2DM, who attended the clinic between January 2019 and May 2024 were initially screened. Using systematic random sampling (1:4), every fourth patient record was selected, resulting in 2010 records. Of these, approximately 50% (1020 patients) had at least 2 years of cumulative follow-up and were included for further review.

Among these, 95.2% had at least one HbA_1c_ record, 47.1% had two, and 39.5% had three or more HbA_1c_ records beyond the baseline. Of the 403 patients (39.5%) with three or more HbA_1c_ records, 202 were documented as having been prescribed a statin.

From this group, 51 patients who met the inclusion criteria were randomly selected as the exposed group (statin users). They were matched to 71 unexposed patients (nonstatin users) at a ratio of ~1:1.4 based on key baseline characteristics. Matching variables included age (± 5 years), BMI, baseline HbA_1c_, blood pressure, and presence of comorbidities such as hypertension. Matching was performed manually using the patients' database, with efforts made to ensure similarity of variables across groups. Balance between the two groups was assessed by descriptive statistics prior to follow-up. All participants were followed for 24 months, with data extracted at baseline and at intervals of 1–3, 4–6, 7–12, 13–18, and 19–24 months after initiation of statin therapy.

### 2.4. Data Collection

Data collection was conducted using SurveyCTO v2.80, an open-source digital tool designed for both mobile and computer-based data collection. The designed questionnaire was uploaded and administered by trained personnel. Participants' hospital registration numbers were used to retrieve electronic medical records. This included demographics (age, sex, marital status, education level, employment, and place of residency), anthropometric data (weight, height, and BMI), vital signs (blood pressure and blood glucose), and clinical history, including family and social risk factors (alcohol use, smoking, and family history of DM).

T2DM-related data—such as duration of disease, antidiabetic medications, statin therapy details (type, dose, and duration of therapy), and comorbidities (hypertension and obesity) were also extracted. Laboratory parameters of interest included HbA_1c_, serum LDL-C, total cholesterol, HDL-C, and serum creatinine.

Baseline data were recorded prior initiation of statin therapy. Follow-up measurements were subsequently extracted at defined intervals post-initiation: 1–3, 4–6, 7–12, 13–18, and 19–24 months.

### 2.5. Exposure and Outcome

The primary exposure variable was initiation of statin therapy. The primary outcome was worsening glycemic control defined as an increase in HbA_1c_ of ≥ 0.2% at 1–3, 7–12, and 19–24 months from baseline [31].

Secondary outcomes were (1) change in HbA_1c_ in relation to statin type, dose, and duration, (2) predictors of ≥ 0.2% rise in HbA_1c_, and (3) proportion of patients who experienced ≥ 0.2% rise in HbA_1c_ at 1–3 and 7–12 months.

### 2.6. Data Processing and Analyzing

After inspection for completeness, the collected data were cleaned, edited, coded, and exported in Excel format. Data were then analyzed using STATA Version 17 and SPSS Version 26. Due to substantial missing data, follow-up intervals at 4–6 and 13–18 months were excluded from the analysis. Data normality was assessed using visual inspection of histograms, measure of central tendency and dispersion, and *Q*–*Q* plots.

Continuous variables were summarized as means (± SD) and median with interquartile range (IQR). Categorical variables were summarized as frequencies and percentages. Baseline sociodemographic and clinical characteristics between statin users and nonusers were compared using independent sample *t*-tests for continuous variables and chi-square tests for categorical variables.

HbA_1c_ levels were compared within and between groups (statin users vs. nonusers; then atorvastatin vs. rosuvastatin vs. nonusers) at baseline, 1–3, 7–12, and 19–24 months using a mixed analysis of variance (ANOVA), with Games–Howell post hoc tests to adjust for unequal variances. Thereafter, we assessed the difference in mean difference (MD) between statin users and nonusers by independent sample *t*-test.

The risk of a ≥ 0.2% rise in HbA_1c_ at follow-up intervals between statin users and nonusers was analyzed using binomial regression. Independent variables that were associated with a ≥ 0.2% rise in HbA_1c_ on the bivariate analysis with (*p* < 0.2) were included in the multivariate binomial regression model. These included statin use status, gender, smoking status, BMI, hypertension, employment status, and baseline HbA_1c_. Adjusted risk ratios (aRRs) and 95% confidence intervals (CIs) were calculated for 122 subjects.

## 3. Results

### 3.1. Enrollment of Participants

A total of 2010 files were selected using systematic random sampling (1:4), selecting every fourth patient who attended clinic. Of these, approximately 50% (*n* = 1020) had two years of cumulative follow-up data and were further reviewed. Among them, 39.5% (*n* = 403) had three HbA_1c_ measurements beyond baseline. Of these, 202 patients had documentation of statin prescription in their medical records.

**Figure 1:**
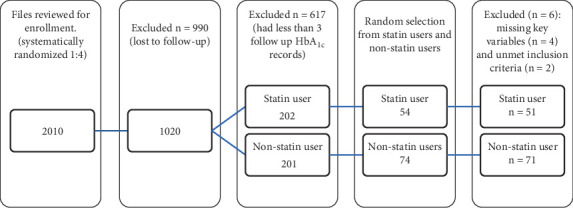
Flow chart for selection of study participant.

The final analytical sample included 122 subjects of the initial 128 recruited. Six participants were excluded due to missing information on key variables (*n* = 4) and failure to meet the inclusion criteria (*n* = 2). In the final sample, 51 participants were randomly selected as statin users and matched to 71 nonstatin users (Figure 1).

### 3.2. Sociodemographic Characteristics

Overall, the study had 71.3% females, with a mean age of 61 ± 10 years, 74.6% were married, 37.7% were retired, and 78.7% of all patients lived in an urban area. Statin prescription was significantly associated with employment status (Table 1).

### 3.3. Baseline Clinical Characteristics

At baseline, 86 patients (70.5%) were on oral hypoglycemic agents; 55.6% of 36 insulin users were on statins, 77% of all participants had hypertension, and 51% of obese participants were statin users. Statins users had baseline HbA_1c_ of 10.6% ± 2.7%, not significantly different from nonstatin users who had HbA_1c_ of 11.3% ± 2.8%. However, lipid profile differed: statin users had higher LDL-C of 3.5 ± 1.1 versus 2.8 ± 1.2 mmol/L and total cholesterol of 5.6 ± 1.3 versus 4.8 ± 1.2 mmol/L. Statin use was significantly associated with baseline LDL-C, total cholesterol, insulin therapy, and obesity (Table 2).

### 3.4. Effect of Statin Therapy on Glycemic Control With Duration of Therapy

**Figure 2:**
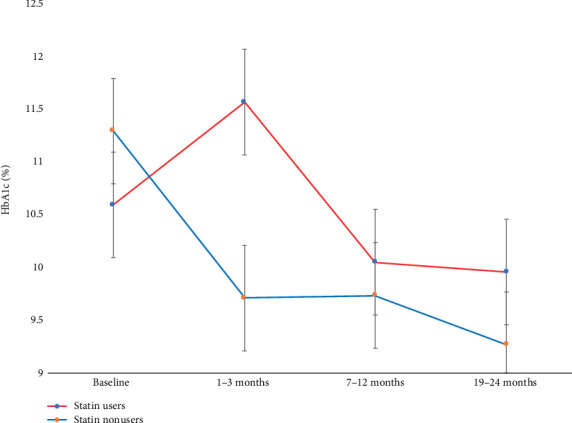
Demonstrating the change of the mean HbA_1c_ for statin users (red line) and nonusers (blue line) over time, at 1–3 months, statin users had an increase of HbA_1c_ of + 0.97% compared to nonusers who had a reduction of − 1.58%, there existed significant mean difference (MD) of 2.56% (95% CI: 3.59–1.53; *p* < 0.001). Nonstatin users had significant and sustained decline in HbA_1c_ from baseline compared to 1–3, 7–12, and 19–24 months (*p* < 0.05).

Among statin users, mean HbA_1c_ increased from 10.6% ± 2.7% at baseline compared to 11.6% ± 2.8% at 1–3 months (*p* = 0.114) thereafter decreased to 10.1% ± 2.2% at 7–12 months (*p* = 1.0), and 10.0% ± 2.5% at 19–24 months (*p* = 1.0). In contrast, nonstatin users had a significant mean HbA_1c_ reduction from 11.3% ± 2.8% at baseline compared to 9.7% ± 2.2% at 1–3 months (*p* = 0.001), 9.7% ± 2.6% at 7–12 months (*p* = 0.011), and 9.3% ± 2.2% at 19–24 months (*p* = 0.001). When comparing the MD of HbA_1c_, significance was observed only at 1–3 months, statin users had an increase of + 0.97% versus nonusers who had a reduction of − 1.58% (MD 2.56%; 95% CI: 1.53–3.59; *p* < 0.001) (Figure 2 and see Table S2 and Figure S1).

Beyond 1–3 months of statin therapy, a chi-square test of independence revealed a significant association between statin use and intensification of antidiabetic medications (*χ*^2^ (1, *N* = 122) = 8.45, *p* = 0.004, *Φ* = 0.26). statin users were more likely to require escalation of diabetes therapy, indicating a possible impact of statins on glycemic control (Table 2).

### 3.5. Effect of Different Types of Statin on Glycemic Control

Examining the three groups (atorvastatin, rosuvastatin, and nonstatin users), patients on atorvastatin (*n* = 46) had a significant increase of mean HbA_1c_ from 10.7% ± 2.8% at baseline compared to 11.9% ± 2.7% at 1–3 months (*p* = 0.04), but thereafter nonsignificant decline to 9.8% ± 2.1% at 7–12 months (*p* = 0.212) and 10.0% ± 2.6% at 19–24 months (*p* = 0.961). In the rosuvastatin group (*n* = 5), HbA_1c_ did not significantly change from 9.5% ± 1.6% at baseline compared to any other points in time. However, nonstatin users (*n* = 71) exhibited significant reduction of mean HbA_1c_ from 11.3% ± 0.4% at baseline to 9.7% ± 0.3% at 1–3 months (*p* < 0.001), 9.7% ± 0.3% at 7–12 months (*p* = 0.001), and 9.3% ± 0.3% at 19–24 months (*p* < 0.001).

**Figure 3:**
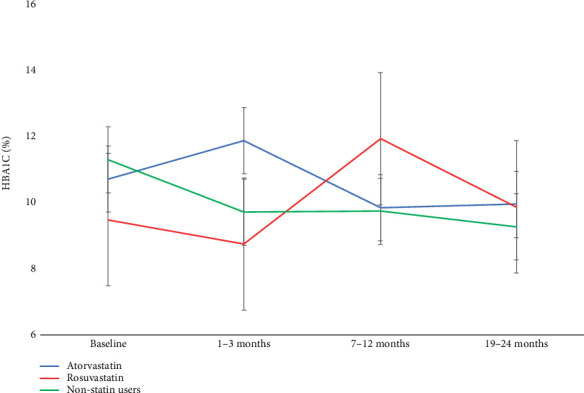
Effect of statins on glycemic control over time: The blue line shows patients on atorvastatin, with a significant mean HbA_1c_ increase from baseline compared to 1–3 months (*p* = 0.04). The red line rosuvastatin group shows no significant reduction from baseline to 1–3 months (*p* = 1.0). The green line (nonstatin users) shows a significant decrease of HbA_1c_ from baseline compared to 1–3 (*p* < 0.001), 7–12 (*p* = 0.001), and 19–24 months (*p* < 0.001).

However, the post hoc comparison using Games–Howell corrections showed nonsignificant difference between nonstatin users and atorvastatin (*p* = 0.243); no other pairwise comparison reached statistical significance (*p* > 0.05). These findings suggest that, although the overall effect was significant, individual group differences were not strong enough to reach significance after correcting for multiple comparisons (Figure 3).

### 3.6. Incidence of ≥ 0.2% Rise in HbA_1c_ Between Statin Users and Nonstatin Users

**Figure 4:**
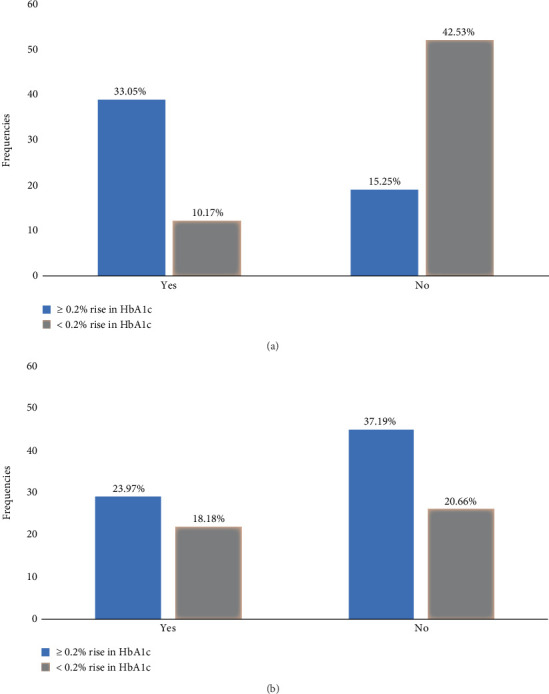
Stratified the cohort into two groups: statin users and nonusers, to observe proportion of a 0.2% change in HbA_1c_. (a) Statin user had higher risk for ≥ 0.2% rise of the mean HbA_1c_ (blue bar) at 1–3 months (*p* < 0.001). (b) Statin users had no significant risk for ≥ 0.2% rise of the mean HbA_1c_ (blue bar) at 7–12 months (*p* = 0.52).

Statin use was significantly associated with a ≥ 0.2% rise in HbA_1c_ at 1–3 months (RR 3.02; 95% CI: 1.97–4.62; *p* < 0.001). But not at 7–12 months (RR 1.23; 95% CI: 0.78 –1.91; *p* = 0.372) (Tables 3 and 4 and Figure 4). At 19–24 months (see Table S1 and Figure S2).

### 3.7. Factors Associated With 0.2% Change in HbA_1c_

Multivariate binomial regression showed statin therapy was significantly associated with ≥ 0.2% rise in HbA_1c_ at 1–3 months, as the only independent risk factor (Table 3). At 7–12 months, female gender and a history of cigarette smoking were significantly associated with a ≥ 0.2% rise in HbA_1c_, while the absence of hypertension significantly reduced likelihood of a ≥ 0.2% rise in HbA_1c_ (Table 4).

## 4. Discussion

The study findings indicate that initiation of statin therapy was associated with a clinically significant rise in HbA_1c_ particularly within the first 3 months. Statin users experienced approximately 1.0% increase in the mean HbA_1c_ within 1–3 months posttherapy. In contrast nonstatin users had a significant and sustained reduction in HbA_1c_ at all time intervals. While the rise in HbA_1c_ among statin users appeared to attenuate over time, it coincided with a higher rate of antidiabetic medication intensification, suggesting a potential statin-related effect on glycemic control.

These findings align closely with a retrospective cohort from the United Kingdom (UK) which reported transient glycemic deterioration among statin users on insulin therapy [39]. Similarly, prospective studies from the Netherlands (CORALL trial), Italy, and Japan observed a modest but significant increase in HbA_1c_ with statin use [40–42]. Notably, the Italian study reported effects persisting up to 12 months, while the CORALL trial involved higher-dose of statin therapy with effects sustained up to 6 months. The difference in dosing and type of statin may explain partially the variation in duration of the effect compared to our study. In contrast, the JUPITER and CARDS trials, identified persistent glycemic effects beyond 24 months, although under more tightly controlled conditions in comparison to our cohort obtained from a primary care setting [15, 17].

The effect of statins on glycemic control in this study was most pronounced with atorvastatin. Patients on atorvastatin had significant increase in mean HbA_1c_ 1–3 months but no sustained increase beyond that point. In contrast, rosuvastatin users did not exhibit any significant change in HbA_1c_ at any point in time, although the sample size for this group was small, limiting interpretability. These findings echo prior studies that suggest atorvastatin may have a more pronounced effect on glycemic parameters compared to other statins potentially due to its a lipophilicity and systemic bioavailability [3, 17, 18, 43].

The mechanistic effect of statin on glycemic control is said to involve impaired insulin sensitivity and/or secretion through several proposed pathways. These include reduced expression of GLUT-2 and GLUT-4 transporters, impaired pancreatic *β*-cell function and altered adipokine profiles [44, 45]. Furthermore, statins may influence hepatic glucose output and systemic inflammation, both of which can affect glycemic control. However, the observed attenuation of the HbA_1c_ rise over time may indicate physiological adaptation or compensatory therapy adjustments.

While direct mechanistic exploration was beyond this study's scope, we observed an association between worsening of glycemic control, higher BMI, and greater insulin use among statin users. Preclinical data suggest that statins may alter adipokine profiles—reducing leptin levels and increasing appetite—potentially contributing to weight gain and hyperglycemia (the so-called “gluttony statins” phenomenon)” [40, 46]. Ongoing research into statin-mediated effects on pancreatic *β*-cell function and skeletal muscle insulin signaling could offer further biological plausibility.

We further assessed the incidence of a ≥ 0.2% increase in mean HbA_1c_ following statin initiation. At 1–3 months, statin users exhibited more than threefold higher risk of experiencing this increment compared with nonusers, even after adjusting for confounders (Table 3). This finding is clinically relevant, particularly given the well-established relationship between elevated HbA_1c_ and cardiovascular as well as microvascular complications [47]. At 7–12 months, statin use was no longer a significant predictor of a ≥ 0.2% rise in HbA_1c_. Instead, female sex and a history of cigarette smoking were independently associated with a ≥ 0.2% rise in HbA_1c_, while the absence of hypertension appeared to be protective. These associations suggest the unmasking of traditional risk factors, similar to other studies [48, 49], following the intensification of antidiabetic medication. These findings may point to underlying metabolic or hormonal and social factors affecting glucose homeostasis.

The larger magnitude of change in mean HbA_1c_ observed in our study is consistent with findings from other observational cohorts done in routine clinical settings [39, 40, 42]. However, a meta-analysis of nine RCTs with an average follow up of 3.6 years reported a smaller change in mean HbA_1c_ among statin users of 0.12%, which may be attributed to the controlled nature of RCTs [50].

The study had several strengths supporting its findings and their application in clinical practice. One notable strength was the use of real-world data, collected through rigorous methods, relying on standardized HbA_1c_ measurements that reflect long-term glycemic control and fewer day-to-day perturbations during stress, illness, or changes in nutrition [10]. Secondly, matching of statin users to nonusers helped minimize potential confounders, and outcomes were assessed across multiple time points to evaluate temporal patterns. The study also enriches the sparse data on statin–diabetes interactions in Tanzania East Africa, providing locally relevant evidence to inform practice.

Nonetheless, the retrospective design limited assessment of actual statin adherence. Approximately 95% of patients at our clinic were prescribed the same type and dosage of statin; moreover, the small sample size of rosuvastatin users precluded strong conclusion about comparisons between statin types. Finally, the single-centered nature of the study and the potential for selection bias could limit the generalizability of the findings.

## 5. Conclusion

The study findings highlight the importance of monitoring glycemic levels (HbA_1c_) for the first 3 months, which appears to decline over time—likely due to intensification of antidiabetic therapy. Atorvastatin may contribute more to this effect than rosuvastatin, though further research is warranted. Clinicians should remain vigilant about glycemic control in the early phase of statin therapy and consider timely therapeutic adjustments where necessary. Larger prospective studies are needed to confirm these findings and explore underlying mechanisms to optimize both cardiovascular and glycemic outcomes associated with statin therapy.

## Figures and Tables

**Table 1:** Baseline sociodemographic characteristics (*N* = 122).

**Variables**	**n** ** (%)**			**p** ** value**
	**Total (** **n** = 122**)**	**Nonstatin (** **n** = 71**)**	**Statin (** **n** = 51**)**	
Gender				0.51
Male	35 (28.7)	22 (31.0)	13 (25.5)	
Female	87 (71.3)	49 (69.0)	38 (74.5)	
Age (years), mean (±SD)	61 (10)	60 (11)	62 (9)	0.175
Marital status				0.38
Single	6 (4.9)	5 (7.0)	1 (2.0)	
Married	91 (74.6)	53 (74.7)	38 (74.5)	
Divorced/widowed	25 (20.5)	13 (18.3)	12 (23.5)	
Highest level of education				0.058
Primary	30 (24.6)	23 (32.4)	7 (13.7)	
Secondary	33 (27.0)	18 (25.4)	15 (29.4)	
University	59 (48.4)	30 (42.2)	29 (56.9)	
Employment status				0.019
Unemployed	11 (9.0)	11 (15.5)	0 (0)	
Employed	33 (27.1)	20 (28.2)	13 (25.5)	
Self-employed	32 (26.2)	15 (21.1)	17 (33.3)	
Retired	46 (37.7)	25 (35.2)	21 (41.2)	
Residency				0.61
Urban	96 (78.7)	57 (80.3)	39 (76.5)	
Rural	26 (21.3)	14 (19.7)	12 (23.5)	
Alcohol use in years (IQR)	25 (20–40)	30 (20–40)	25 (20–40)	0.59
History of cigarette smoking				0.67
No	116 (95.1)	67 (94.4)	49 (96.1)	
Yes	6 (4.9)	4 (5.6)	2 (3.9)	

Abbreviations: IQR, interquartile range; SD, standard deviation.

**Table 2:** Baseline clinical characteristics (*N* = 122).

**Variables**	**Mean (±SD)**			**p** ** value**
	**Total (** **n** = 122**)**	**Nonstatin (** **n** = 71**)**	**Statin (** **n** = 51**)**	
Baseline laboratory values				
HbA_1c_ (%)	11.0 (3.5)	11.3 (2.8)	10.6 (2.7)	0.249
LDL-C (mmol/L)	3.1 (1.2)	2.8 (1.2)	3.5 (1.1)	0.010
HDL-C (mmol/L)	1.4 (0.9)	1.5 (0.3)	1.2 (1.3)	0.169
Creatinine (*μ*mol/L)	75.3 (28.8)	75.3 (29.7)	75.2 (27.7)	0.985
Total cholesterol (mmol/L)	5.2 (1.3)	4.8 (1.2)	5.6 (1.3)	0.001
Baseline anthropometric values				
Systolic BP (mmHg)	142.6 (23.4)	140.8 (23.38)	145.0 (23.4)	0.333
Diastolic BP (mmHg)	83.8 (11.3)	84.3 (10.3)	83.2 (12.6)	0.613
BMI (kg/m^2^), mean (±SD)	28.9 (5.1)	27.8 (4.6)	30.3 (5.3)	0.008
Underweight	1 (0.8)	1 (1.4)	0 (0)	
Normal	13 (10.7)	9 (12.7)	4 (7.8)	
Overweight	66 (54.1)	45 (63.4)	21 (41.2)	
Obese	42 (34.4)	16 (22.5)	26 (51.0)	0.013
DM medications (*n* (%))				
Oral	86 (70.5)	55 (77.5)	31 (60.8)	
Insulin	36 (29.5)	16 (22.5)	20 (39.2)	0.046
Anti-DM intensification (*n* (%))^‡^				
No	62 (50.8)	44 (62)	18 (35.3)	
Yes	60 (49.2)	27 (38)	33 (64.7)	0.004
Hypertension (*n* (%))				
No	28 (23.0)	22 (79.0)	6 (21.0)	
Yes	94 (77.0)	49 (69.0)	45 (88.2)	0.013
Duration of T2DM	13.5 (7.3)	13.2 (7.4)	14.0 (7.3)	0.562
Obesity (*n* (%))				
No	80 (65.6)	55 (77.5)	25 (49.0)	
Yes	42 (34.4)	16 (22.5)	26 (51.0)	0.028

Abbreviations: BMI, body mass index; BP, blood pressure; DM, diabetes mellitus; HbA_1c_, glycated hemoglobin; HDL-C, high-density lipoprotein cholesterol; LDL-C, low-density lipoprotein cholesterol.

^‡^At 1–3 months and beyond.

**Table 3:** Results of binomial regression of factors associated with ≥ 0.2% rise in HbA_1c_ 1–3 months from baseline (*N* = 122).

**Variables**	**≥ 0.2% rise in HbA**_**1c**_ ** (** **n** ** (%))**	**RR (95% CI)**	**p** ** value**	**aRR (95% CI)**	**p** ** value**
Statin therapy					
No	18 (25.35)	Ref			
Yes	39 (76.47)	3.02 (1.97–4.62)	< 0.001	3.24 (2.13–4.94)	< 0.001
Gender					
Male	17 (48.57)	Ref			
Female	40 (45.98)	0.95 (0.63–1.43)	0.793	—	—
Marital status					
Single	2 (33.3)	Ref			
Married	43 (47.25)	1.42 (0.45–4.49)	0.553	—	—
Divorced/widowed	12 (48.0)	1.44 (0.43–4.79)	0.552	—	—
Employment status					
Unemployed	8 (72.7)	Ref			
Employed	14 (42.4)	0.7 (0.21–1.57)	0.276	—	—
Self-employed	12 (37.5)	1.2 (0.79–1.83)	0.387	—	—
Retired	24 (52.2)	1.31 (0.87–1.96)	0.294	—	—
Residency					
Rural	12 (46.15)	Ref			
Urban	45 (46.88)	1.02 (0.64–1.62)	0.948	—	—
History of cigarette smoking					
No	53 (45.69)	Ref			
Yes	4 (66.67)	1.46 (0.8–2.66)	0.217	—	—
Baseline BMI					
Normal	6 (46.15)	Ref			
Overweight	30 (45.45)	1.06 (0.55–2.05)	0.861	—	—
Obese	21 (50.0)	1.17 (0.59–2.29)	0.655	—	—
Hypertension					
No	12 (42.86)	Ref			
Yes	45 (47.87)	1.12 (0.693–1.8)	0.649	—	—
Anti-DM intensification					
No	34 (54.8)	Ref			
Yes	24 (40.0)	1.27 (0.87–1.86)	0.215	—	—
Total cholesterol (mmol/L)					
≤ 5.2	19 (45.24)	Ref			
> 5.2	38 (47.5)	1.05 (0.7–1.57)	0.813	—	—

Abbreviations: aRR, adjusted risk ratio; BMI, body mass index; HbA_1c_, glycated hemoglobin; RR, relative risk.

**Table 4:** Results of binomial regression of factors associated with ≥ 0.2% rise in HbA_1c_ 7–12 months from baseline (*N* = 122).

**Variables**	**≥ 0.2% rise in HbA**_**1c**_ ** (** **n** ** (%))**	**RR (95% CI)**	**p** ** value**	**aRR (95% CI)**	**p** ** value**
Statin therapy					
No	25 (35.21)	Ref			
Yes	22 (43.14)	1.23 (0.78–1.91)	0.372		
Gender					
Male	9 (25.71)	Ref			
Female	38 (43.68)	1.69 (0.92–3.13)	0.09	2.27 (1.44–3.58)	< 0.001
Marital status					
Single	2 (33.3)	Ref			
Married	36 (39.56)	1.19 (0.37–3.78)	0.772	—	—
Divorced/widowed	9 (36.0)	1.08 (0.31–3.76)	0.904	—	
Employment status					
Unemployed	3 (27.27)	Ref			
Employed	17 (51.52)	1.89 (0.68–5.24)	0.222	—	—
Self-employed	11 (34.38)	1.26 (0.43–3.7)	0.674	—	—
Retired	16 (34.78)	1.28 (0.45–3.62)	0.648	—	—
Residency					
Rural	10 (38.46)	Ref			
Urban	37 (38.54)	1.0 (0.58–1.73)	0.994	—	—
History of cigarette smoking					
No	43 (37.07)	Ref			
Yes	4 (66.67)	1.8 (0.97–3.32)	0.061	3.34 (2.32–4.82)	< 0.001
Baseline BMI class					
Normal	4 (28.57)	Ref			
Overweight	23 (34.85)	1.22 (0.5–2.97)	0.662	—	—
Obese	20 (47.62)	1.67 (0.69–4.05)	0.259	—	—
Comorbidities except HTN					
No	10 (52.63)	Ref			
Yes	37 (35.92)	0.68 (0.41–1.12)	0.133	0.47 (0.30–0.74)	< 0.001
Hypertension					
No	14 (50)	Ref			
Yes	33 (35.11)	0.7 (0.44–1.11)	0.367	—	—
Anti-DM intensification					
No	34 (54.8)	Ref			
Yes	34 (56.7)	1.02 (0.65–1.6)	0.92	—	—
Total cholesterol (mmol/L)					
≤ 5.2	16 (38.1)	Ref			
> 5.2	31 (38.75)	1.02 (0.63–1.63)	0.944	—	—

Abbreviations: aRR, adjusted risk ratio; BMI, body mass index; HbA_1c_, glycated hemoglobin; HTN, hypertension; RR, relative risk.

## Data Availability

The data that support the findings of this study are available from the corresponding author upon reasonable request.

## References

[1] Sehra D., Sehra S., Sehra S. T. (2017). Cardiovascular Pleiotropic Effects of Statins and New Onset Diabetes: Is There a Common Link: Do We Need to Evaluate the Role of KATP Channels?. *Expert Opinion on Drug Safety*.

[2] Blais J. E., Wei Y., Yap K. K. (2021). Trends in Lipid-Modifying Agent Use in 83 Countries. *Atherosclerosis*.

[3] Alvarez-Jimenez L., Morales-Palomo F., Moreno-Cabañas A., Ortega J. F., Mora-Rodríguez R. (2023). Effects of Statin Therapy on Glycemic Control and Insulin Resistance: A Systematic Review and Meta-Analysis. *European Journal of Pharmacology*.

[4] Bellia A., Rizza S., Lombardo M. F. (2012). Deterioration of Glucose Homeostasis in Type 2 Diabetic Patients One Year After Beginning of Statins Therapy. *Atherosclerosis*.

[5] Liew S. M., Lee P. Y., Hanafi N. S. (2014). Statins Use Is Associated With Poorer Glycaemic Control in a Cohort of Hypertensive Patients With Diabetes and Without Diabetes. *Diabetology and Metabolic Syndrome*.

[6] Fong C. W. (2014). Statins in Therapy: Understanding their Hydrophilicity, Lipophilicity, Binding to 3-Hydroxy-3-Methylglutaryl-CoA Reductase, Ability to Cross the Blood Brain Barrier and Metabolic Stability Based on Electrostatic Molecular Orbital Studies. *European Journal of Medicinal Chemistry*.

[7] Endo A. (2008). A Gift From Nature: The Birth of the Statins. *Nature Medicine*.

[8] Endo A. (2010). A Historical Perspective on the Discovery of Statins. *Proceedings of the Japan Academy Series B: Physical and Biological Sciences*.

[9] Liao J. K., Laufs U. (2005). Pleiotropic Effects of Statins. *Annual Review of Pharmacology and Toxicology*.

[10] ADA (2022). Standards of Medical Care in Diabetes—2022Abridged for Primary Care Providers. *Clinical Diabetes*.

[11] Arnett D. K., Blumenthal R. S., Albert M. A. (2019). 2019 ACC/AHA Guideline on the Primary Prevention of Cardiovascular Disease: Executive Summary: A Report of the American College of Cardiology/American Heart Association Task Force on Clinical Practice Guidelines. *Circulation*.

[12] Clough J. D., Martin S. S., Navar A. M. (2019). Association of Primary Care Providers’ Beliefs of Statins for Primary Prevention and Statin Prescription. *Journal of the American Heart Association*.

[13] Freeman D. J., Norrie J., Sattar N. (2001). Pravastatin and the Development of Diabetes Mellitus: Evidence for a Protective Treatment Effect in the West of Scotland Coronary Prevention Study. *Circulation*.

[14] Beckett R. D., Schepers S. M., Gordon S. K. (2015). Risk of New-Onset Diabetes Associated With Statin Use. *SAGE Open Medicine*.

[15] Livingstone S. J., Looker H. C., Akbar T. (2016). Effect of Atorvastatin on Glycaemia Progression in Patients With Diabetes: An Analysis From the Collaborative Atorvastatin in Diabetes Trial (CARDS). *Diabetologia*.

[16] Sunjaya A. P., Sunjaya A. F., Halim S., Ferdinal F. (2018). Risk and Benefits of Statins in Glucose Control Management of Type II Diabetes. *International Journal of Angiology*.

[17] Ridker P. M., Pradhan A., MacFadyen J. G., Libby P., Glynn R. J. (2012). Cardiovascular Benefits and Diabetes Risks of Statin Therapy in Primary Prevention: An Analysis From the JUPITER Trial. *Lancet*.

[18] Cui J. Y., Zhou R. R., Han S., Wang T. S., Wang L. Q., Xie X. H. (2018). Statin Therapy on Glycemic Control in Type 2 Diabetic Patients: A Network Meta-Analysis. *Journal of Clinical Pharmacy and Therapeutics*.

[19] US FDA (2012). *US Food and Drug Administration (FDA). Drugs: FDA Safety Communication: Important Safety Label Change*.

[20] Ozen G., Dell’Aniello S., Pedro S., Michaud K., Suissa S. (2023). Reduction of Cardiovascular Disease and Mortality Versus Risk of New-Onset Diabetes Mellitus With Statin Use in Patients With Rheumatoid Arthritis. *Arthritis Care & Research*.

[21] Bierman E. L. (1992). George Lyman Duff Memorial Lecture Atherogenesis in Diabetes. *Arteriosclerosis and Thrombosis: A Journal of Vascular Biology*.

[22] Guo J., Jiang Z., Xia Y., Wang H., Tang Q., Meng B. (2024). The Association Between Statin Use and Diabetic Nephropathy in US Adults: Data From NHANES 2005-2018. *Frontiers in Endocrinology*.

[23] Tonelli M., Keech A., Shepherd J. (2005). Effect of Pravastatin in People With Diabetes and Chronic Kidney Disease. *Journal of the American Society of Nephrology*.

[24] Stratton I. M., Adler A. I., Neil A. W. (2000). Association of Glycaemia With Macrovascular and Microvascular Complications of Type 2 Diabetes (UKPDS 35): Prospective Observational Study. *BMJ*.

[25] Ogurtsova K., Guariguata L., Barengo N. C. (2022). IDF Diabetes Atlas: Global Estimates of Undiagnosed Diabetes in Adults for 2021. *Diabetes Research and Clinical Practice*.

[26] Stanifer J. W., Cleland C. R., Makuka G. J. (2016). Prevalence, Risk Factors, and Complications of Diabetes in the Kilimanjaro Region: A Population-Based Study From Tanzania. *PLoS One*.

[27] Zhang L., He S., Li Z. (2019). Apolipoprotein e Polymorphisms Contribute to Statin Response in Chinese ASCVD Patients With Dyslipidemia. *Lipids in Health and Disease*.

[28] Masemola M. L., Alberts M., Urdal P. (2007). Apolipoprotein E Genotypes and Their Relation to Lipid Levels in a Rural South African Population 1. *Scandinavian Journal of Public Health*.

[29] Hassanein M., Shafi T. (2022). Assessment of Glycemia in Chronic Kidney Disease. *BMC Medicine*.

[30] Schachter M. (2005). Chemical, Pharmacokinetic and Pharmacodynamic Properties of Statins: An Update. *Fundamental and Clinical Pharmacology*.

[31] Khaw K.-T., Wareham N., Luben R. (2001). Glycated Haemoglobin, Diabetes, and Mortality in Men in Norfolk Cohort of European Prospective Investigation of Cancer and Nutrition (EPIC-Norfolk). *BMJ*.

[32] Johnston K. M., Lakzadeh P., Donato B. M. K., Szabo S. M. (2019). Methods of Sample Size Calculation in Descriptive Retrospective Burden of Illness Studies. *BMC Medical Research Methodology*.

[33] Ntinginya N. E., te Brake L., Sabi I. (2022). Rifapentine and Isoniazid for Prevention of Tuberculosis in People With Diabetes (PROTID): Protocol for a Randomised Controlled Trial. *Trials*.

[34] James P. A., Oparil S., Carter B. L. (2014). 2014 Evidence-Based Guideline for the Management of High Blood Pressure in Adults. *Jama*.

[35] Stevens P. E., Ahmed S. B., Carrero J. J. (2024). KDIGO 2024 Clinical Practice Guideline for the Evaluation and Management of Chronic Kidney Disease. *Kidney International*.

[36] Pi-Sunyer F. X., Becker D. M., Bouchard C. (1998). Clinical Guidelines on the Identification, Evaluation, and Treatment of Overweight and Obesity in Adults: Executive Summary. *American Journal of Clinical Nutrition*.

[37] Kim C., Bullard K. M. K., Herman W. H., Beckles G. L. (2010). Association Between Iron Deficiency and A1C Levels Among Adults Without Diabetes in the National Health and Nutrition Examination Survey, 1999-2006. *Diabetes Care*.

[38] McAvoy N., Thomson E., Wilson E. S. (2012). Hepatic Failure. *Anaesthesia and Intensive Care Medicine*.

[39] Anyanwagu U., Mamza J., Donnelly R., Idris I. (2017). Effects of Background Statin Therapy on Glycemic Response and Cardiovascular Events Following Initiation of Insulin Therapy in Type 2 Diabetes: A Large UK Cohort Study. *Cardiovascular Diabetology*.

[40] Bardini G., Giannini S., Rotella C. M., Pala L., Cresci B., Mannucci E. (2016). Lower and Higher-Potency Statins on Glycemic Control in Type 2 Diabetes: A Retrospective Cohort Study. *Diabetes Research and Clinical Practice*.

[41] Simsek S., Schalkwijk C. G., Wolffenbuttel B. H. R. (2012). Effects of Rosuvastatin and Atorvastatin on Glycaemic Control in Type 2 Diabetes-the CORALL Study. *Diabetic Medicine*.

[42] Takano T., Yamakawa T., Takahashi M., Kimura M., Okamura A. (2006). Influences of Statins on Glucose Tolerance in Patients With Type 2 Diabetes Mellitus. *Journal of Atherosclerosis and Thrombosis*.

[43] Kim D. W., Kim D. H., Park J. H. (2019). Association Between Statin Treatment and New-Onset Diabetes Mellitus: A Population-Based Case-Control Study. *Diabetology and Metabolic Syndrome*.

[44] Corrao G., Ibrahim B., Nicotra F. (2014). Statins and the Risk of Diabetes: Evidence From a Large Population-Based Cohort Study. *Diabetes Care*.

[45] Galicia-Garcia U., Jebari S., Larrea-Sebal A. (2020). Statin Treatment-Induced Development of Type 2 Diabetes: From Clinical Evidence to Mechanistic Insights. *International Journal of Molecular Sciences*.

[46] Singh P., Zhang Y., Sharma P. (2018). Statins Decrease Leptin Expression in Human White Adipocytes. *Physiological Reports*.

[47] Menon V., Kumar A., Patel D. R. (2020). Impact of Baseline Glycemic Control on Residual Cardiovascular Risk in Patients With Diabetes Mellitus and High-Risk Vascular Disease Treated With Statin Therapy. *Journal of the American Heart Association*.

[48] Dawite F., Girma M., Shibiru T. (2023). Factors Associated With Poor Glycemic Control Among Adult Patients With Type 2 Diabetes Mellitus in Gamo and Gofa Zone Public Hospitals, Southern Ethiopia: A Case-Control Study. *PLoS One*.

[49] Fina Lubaki J. P., Omole O. B., Francis J. M. (2022). Glycaemic Control Among Type 2 Diabetes Patients in Sub-Saharan Africa From 2012 to 2022: A Systematic Review and Meta-Analysis. *Diabetology and Metabolic Syndrome*.

[50] Erqou S., Lee C. C., Adler A. I. (2014). Statins and Glycaemic Control in Individuals With Diabetes: A Systematic Review and Meta-Analysis. *Diabetologia*.

