# Supplementary material for: Effects of Statin Therapy on Glycemic Control and Associated Factors Among Type 2 Diabetes Mellitus Patients in Northeastern Tanzania: A Retrospective Cohort Study
Source: J Diabetes Res. 2025 Aug 1;2025:6626154. doi: 10.1155/jdr/6626154 (PMC12334290; doi:10.1155/jdr/6626154)
Supplement: Supporting Information — Additional supporting information can be found online in the Supporting Information section. Additional information has been uploaded on the additional information section. [file 6626154.f1.docx]

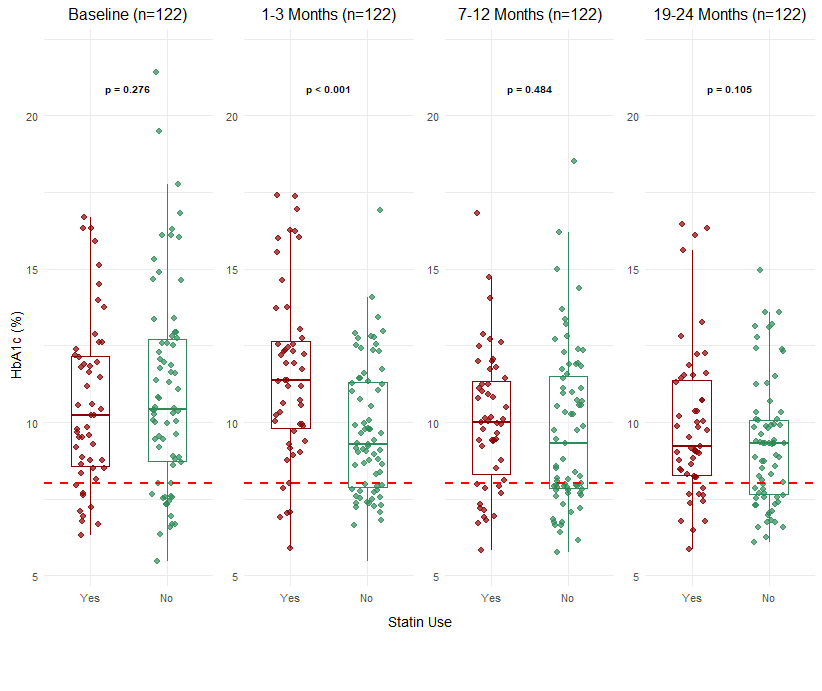


**Supplementary figure 1 with individual data points.** Demonstrating the change of the mean HbA_1c_ for statin users (red box-plot) & non-users (green box-plot) over time, at 1–3 months statin users had an increase of HbA_1c_ of + 0.97% compared to non-users who had a reduction of -1.58%, there existed significant mean difference (MD) of 2.56% (95% CI; 3.59 - 1.53; *p* < 0.001). Non statin users had significant and sustained decline in HbA_1c_ from baseline compared to 1–3 months, 7–12 months to 19–24 months (p < 0.05).

**NOTE:** Generated by R.

The difference in the mean difference at 7–12 months for statin user had reduction in HbA_1c_ of -0.54% vs. -1.56% for non-user MD of -2.1 (95% CI; -2.28 to 0.25; *p* = 0.116) and at 19–24 of -0.66% vs. -2.03% for non-users MD of -2.69% (95% CI; -2.73 to -0.001; *p* = 0.050)

**
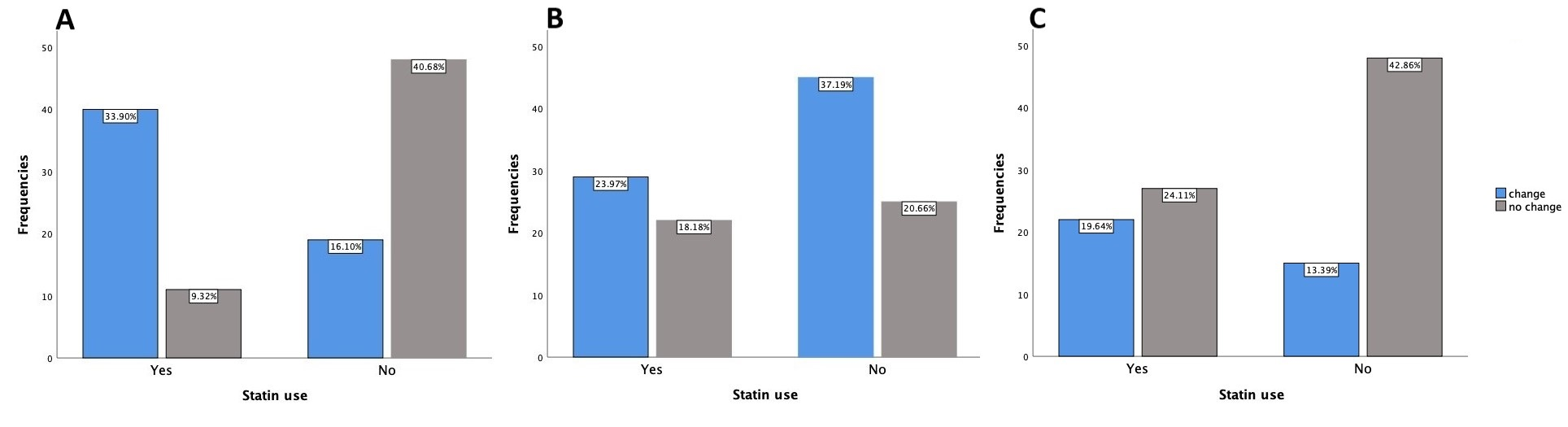
**

**Supplementary Figure 2.** Stratified the cohort into two groups; statin users and non-users, to observe proportion of a 0.2% change in HbA_1c_, **A**; statin user had higher risk ≥ 0.2% rise of the mean HbA_1c_ (blue bar) at 1–3 months (*p* < 0.001). **B**; statin users had no significant risk for risk ≥ 0.2% rise of the mean HbA_1c_ (blue bar) at 7–12 months (*p* = 0.520). **C**; statin users had a risk of ≥ 0.2% rise of the mean HbA_1c_ (blue bar) at 19–24 months (*p* = 0.012)

**Incidence of 0.2% rise in HbA_1c_between statin users and non-statin users**

Statin use was significantly association with 0.2% rise in HbA_1c_ at 19–24 months (RR 1.88; 95% CI: 1.13–3.15; *p* = 0.016). Moreover, female gender was also appeared to be significant but after adjustments the statistical significance was borderline at p = 0.056.

**Supplementary Table 1**: Results of binomial regression of factors associated with 0.2% rise in HbA1c 19–24 months from baseline (N=122)

| Variables | 0.2% HbA_1c_ rise  (n (%)) | RR (95% CI) | p-value | aRR (95% CI) | p-value |
| --- | --- | --- | --- | --- | --- |
| Statin therapy |  |  |  |  |  |
| No | 17 (23.94) | Ref |  |  |  |
| Yes | 23 (45.1) | 1.88 (1.13–3.15) | 0.016 | 1.89 (1.15–3.11) | 0.012 |
| Gender |  |  |  |  |  |
| Male | 6 (17.14) | Ref |  |  |  |
| Female | 34 (39.08) | 2.28 (1.05–4.94) | 0.037 | 2.12 (0.98–4.59) | 0.056 |
| Marital status |  |  |  |  |  |
| Single | 1 (16.67) | Ref |  |  |  |
| Married | 31 (34.07) | 1.42 (0.45–4.49) | 0.553 | - | - |
| Divorced/Widowed | 8 (32.0) | 1.44 (0.43–4.79) | 0.552 | - | - |
| Employment status |  |  |  |  |  |
| Unemployed | 0 (18.18) | ref |  |  |  |
| Employed | 14 (42.42) | 1.15 (0.66–1.99) | 0.622 | - | - |
| Self employed | 9 (28.12) | 0.76 (0.39–1.49) | 0.425 | - | - |
| Residency |  |  |  |  |  |
| Rural | 7 (26.92) | Ref |  |  |  |
| Urban | 33 (34.38) | 1.28 (0.64–2.55) | 0.488 | - | - |
| History of cigarette smoking |  |  |  |  |  |
| No | 38 (32.76) | Ref |  |  |  |
| Yes | 2 (33.33) | 1.02 (0.32–3.25) | 0.977 | - | - |
| Hypertension |  |  |  |  |  |
| No | 9 (32.14) | Ref |  |  |  |
| Yes | 31 (32.98) | 1.03 (0.693–1.8) | 0.934 | - | - |
| Anti-DM intensification |  |  |  |  |  |
| No | 33 (53.2) | Ref |  |  |  |
| Yes | 40 (66.7) | 0.64 (0.38–1.09) | 0.101 | - | - |
|  |  |  |  |  |  |

BMI: Body mass index; HbA_1c_: Glycated hemoglobin; LDL-C: Low-density lipoprotein cholesterol; RR: Relative risk; aRR: adjusted RR.

**Average Treatment Effect (ATE) of Statins On glycemic control**

We further assessed the risk of poor glycemic control in our study by using augmented inverse probability weighting (AIPW) with Poisson regression Model, assessing the ATE at 1–3 months. Statin use was significantly associated with an increased rate of poor glycemic control, 67% higher rate of poor glycemic control among statin users compared to non-users.

**Supplementary table 2.** Association between statin use and risk of poor glycemic control: AIPW Poisson Model Estimates

| Effect type | Comparison | Coefficient | Std. Err. | z | p-value | (95% CI) |
| --- | --- | --- | --- | --- | --- | --- |
| ATE | Yes vs No (Statin) | 0.515 | 0.085 | 6.04 | <0.001 | [0.348, 0.683] |
| POmean | No (Statin) | 0.226 | 0.50 | 4.49 | <0.001 | [0.127, 0.32] |
|  |  |  |  |  |  |  |

ATE: Average treatment effect; POmean: potential outcome means; Std. Err: Standard Error; z: z-value
